# Phosphorylcholine-Functionalized PEDOT-Gated Organic Electrochemical Transistor Devices for Ultra-Specific and Sensitive C-Reactive Protein Detection

**DOI:** 10.3390/polym15183739

**Published:** 2023-09-12

**Authors:** Sihao Qian, Shouyan Zhang, Danni Chen, Jun Wang, Wei Wu, Shuhua Zhang, Zhi Geng, Yong He, Bo Zhu

**Affiliations:** 1 State Key Laboratory for Modification of Chemical Fibers and Polymer Materials, College of Materials Science and Engineering, Donghua University, Shanghai 201620, China; qiansihao@163.com; 2 School of Materials Science and Engineering, Shanghai Engineering Research Center of Organ Repair, Shanghai University, Shanghai 200444, China; zhangshouyan1130@163.com (S.Z.); danny_chen0421@163.com (D.C.); estherwang0525@163.com (J.W.); wu10001300@163.com (W.W.); shzhang1992@shu.edu.cn (S.Z.); gengzhi@shu.edu.cn (Z.G.); 3 Innovation Center for Textile Science and Technology, Donghua University, Shanghai 201620, China

**Keywords:** C-reactive protein, organic electrochemical transistor, zwitterionic phosphorylcholine, antifouling

## Abstract

Affinity-based organic electrochemical transistor (OECT) sensors offer an attractive approach to point-of-care diagnostics due to their extreme sensitivity and easy operation; however, their application in the real world is frequently challenged by the poor storage stability of antibody proteins and the interference from biofouling in complex biofluids. In this work, we developed an antibody-free and antifouling OECT biosensor to detect C-reactive protein (CRP) at ultra-high specificity and sensitivity. The key to this novel biosensor is the gate coated by phosphorylcholine-functionalized poly (3,4-ethylene dioxythiophene) (PEDOT-PC), which possesses large capacitance and low impedance, prevents biofouling of bovine serum albumin (BSA) and the fetal bovine serum (FBS), and interacts specifically with CRP molecules in the presence of calcium ions. This PEDOT-PC-gated OECT biosensor demonstrated exceptional sensitivity when detecting the CRP molecules at 10 pg/mL, while significantly depressing the signal from the nonspecific binding. This indicates that this biosensor could detect the CRP molecules directly without nonspecific binding blocking, the usual process for the earlier transistor sensors before detection. We envision that this PEDOT-PC-gated OECT biosensor platform may offer a potentially valuable tool for point-of-care diagnostics as it alleviates concerns about poor antibody stability and BSA blocking inconstancy.

## 1. Introduction

C-reactive protein (CRP) is a widely used biomarker for differentiating viral infections; it is a non-glycosylated protein with a molecular weight of approximately 115 kDa, composed of five identical spherical subunits non-covalently bound to each other [1]. These subunits form a circular structure known as pentraxin [2]. Measuring the CRP blood level has become routine practice due to its significance as a biomarker for evaluating systemic inflammation resulting from immune system activation, such as infection and tissue damage [3]. An average CRP value in human blood is usually below 10 μg mL^−1^, while in the case of an acute inflammatory stimulus, the concentration of CRP in the blood serum can be dramatically increased by 1000-fold [4]. Moreover, the correlation between CRP levels and prognosis in cancer patients has been well-established. CRP is widely recognized in oncology as a reliable biomarker for assessing survival, cancer risks, and tumor recurrence [5]. CRP concentration also serves as an index to evaluate the risk of cardiovascular disease. It is particularly critical for myocardial infarction, which is strongly associated with CRP levels [6,7]. Therefore, CRP is a valuable biomarker for identifying inflammation and evaluating the risks associated with inflammation-related diseases.

Several chemical and optical techniques have been employed to detect CRP, such as enzyme-linked immunosorbent assay (ELISA), visual agglutination, and immunoturbidimetric [8,9,10]; however, these methods still have some insurmountable drawbacks, including the need for costly equipment, complex sample preparation, extensive labor, strict operational protocols, time-intensive procedures, and indirect detection due to their reliance on optical transduction and the use of labeled reagents [11]. Recently, portable and easy-to-operate transistor-type sensors are attractive for point-of-care testing of CRP in biofluids. An electrolyte-gated field-effect transistor (EGOFET) immunosensor was recently proposed to detect CRP at a high sensitivity [12,13]. In this case, the anti-CRP antibody was physisorbed on a P3HT film bridging source and drain. This transistor-based sensor could reach a detection limit of 2 pM (0.22 ng/mL) and work in a dynamic range spanning six orders of magnitude (from 4 pM to 2 μM). Later, an organic field-effect transistor (OFET) CRP sensor was also developed using a thermally stable organic semiconductor [4]. The sensitivity for detecting CRP antigen down to 1 µg/mL can be achieved by immobilizing anti-CRP monoclonal antibodies on the extended gate. Recently, a fibrous organic electrochemical transistor (OECT) was constructed from boron nitride (FBN)-mediated polypyrrole (PPy) neurofibers for CRP detection. The device’s high surface area and large transconductance guaranteed its low detection limit of 10 pg/mL with good reproducibility [14]. The OECT device’s high sensitivity and easy operation make it an attractive candidate for CRP detection [15]. OECTs feature a significant electrochemical amplification for biosensing as they have a considerable transconductance and a volumetric ionic–electronic coupling in the channel material [16,17,18]. The OECT device can efficiently capture ion fluxes and utilizes this ionic–electronic coupling to convert ionic signals to electronic ones [19,20], making OECTs an efficient ion-to-electron transducer with low impedance, which is critical and appealing for detecting biological markers [19,21,22,23,24]. Previous studies have demonstrated that OECT-based biosensors have a much higher sensitivity to detect protein markers than traditional electrochemical methods thanks to their inherent amplification capability [25,26,27,28]; however, the point-of-care application of OECT biosensing is challenged by two critical issues, i.e., the long-term storage stability of antibody proteins grafted on gates/channels [29], and the interference from biofouling in the complex biofluids [30].

Phosphorylcholine-functionalized polymers have recently gained extensive attention for acting as bioelectrodes in biomedical engineering, as they feature strong biofouling resistance due to their neutrality and superhydrophilicity [31,32]. Notably, the phosphorylcholine group was found to interact specifically with CRP in the presence of calcium ions [33]. It was demonstrated that the phosphorylcholine-functionalized polymers could specifically interact with CRP within a wide dynamic range [34]. Recent studies have further confirmed the specific binding of CRP to the phosphorylcholine groups on conducting polymers in the calcium-containing buffer [35,36,37].

Inspired by these exciting findings, we developed an antibody-free OECT platform to specifically detect CRPs, using the phosphorylcholine-functionalized poly(3,4-ethylene dioxythiophene) (PEDOT-PC) coated electrode as the gate. The PEDOT-PC electrode presented low impedance, superhydrophilicity, and extreme protein resistance. Furthermore, the impact of calcium ion (Ca^2+^) concentration on the interaction with CRP was systematically investigated to discover the optimized Ca^2+^ concentration (1 mM) for the interaction with CRP. The PEDOT-PC gate endows the OECT biosensor with the capability to detect CRP specifically by preventing the nonspecific binding of bovine serum albumin proteins. Taking advantage of the high transconductance of OECTs, as well as the antifouling properties and specific CRP binding capability of PEDOT-PC, this OECT device demonstrated an exceptional sensitivity to detect CRP at levels as low as 10 pg/mL, which was significantly lower than the most detection limits reported for other transistor approaches. We envision that this OECT approach could pave the way toward sensitive and easy-to-operate CRP biosensors for point-of-care applications in the real world.

## 2. Experimental Section

### 2.1. Devices Fabrication

**Figure 1:**
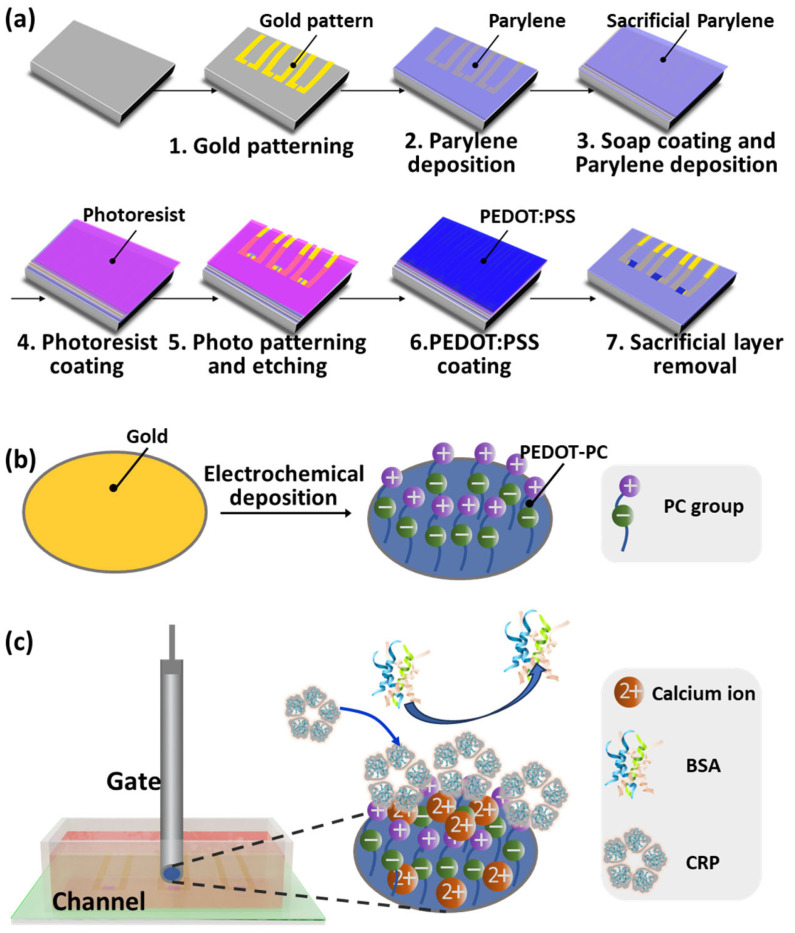
(**a**) Fabrication process flow of the channel of PC-OECT array. (**b**) Preparation of the gate of PC-OECT. (**c**) A schematic diagram of the PC-OECT for specific detection of CRP.

The OECT device was fabricated by a lithography-combined microfabrication process involving the deposition and patterning of the Au pattern, the Parylene packaging, and the fabrication of PEDOT:PSS channels. The overall process flow is shown in Figure 1a. The gold electrodes and interconnecting lanes on a flexible substrate prepared by a conventional lift-off process are described below. The S1813 photoresist (Dow-shipley) was spin-coated on the glass substrate at 3500 rpm, baked at 115 °C for 60 s, exposed to UV light for 2 s through the photomask aligned by a mask aligner (URE2000/25), and developed in the ZX-238 developer (Jianghua Micro-Electronic Materials Co., Ltd., Wuxi, China). Then, the substrate patterned with S1813 photoresist was cleaned by a low-temperature O_2_ plasma processor (SYDT01E, OPS, China) at 400 W for 10 min. It was followed by the deposition of 10 nm of chromium and 160 nm of gold using an electron beam evaporation system (MBE-600). The excess photoresist was washed away by N-methyl-2-pyrrolidone (NMP, Jianghua Micro-Electronic Materials Co., Ltd., China).

After that, Parylene C layer with a thickness of 3 μm was deposited to encapsulate the gold interconnecting lanes from dichloro-[2,2]-paracyclophane (Saen Chemical Technology Co., Ltd., Shanghai, China). The interfacial adhesion was improved by coating 3-(trimethoxysilyl) propyl methacrylate (silane A174, Sigma-Aldrich, Shanghai, China) solution to the underlying substrate before Parylene deposition. Another 3 μm Parylene C layer was deposited onto the passivation layer as a sacrificial layer after a diluted micro 90 industrial cleaner (2% in DI water) was spin-coated onto the underlying encapsulation layer as an antiadhesive layer. The AZ9260 photoresist (Merck, Rahway, NJ, USA) was then spin-coated, baked at 105 °C for 180 s, exposed to UV light for 30 s through a photomask aligned by the mask aligner, and developed in the ZX238 developer. The Parylene C substrates exposed by the photoresist pattern were then reactive-ion etched by the O_2_ plasma in an inductively coupled plasma instrument (ICP-8101, Beijing Chuangshiweina Technology Co., Ltd., Beijing, China) to open contact pads and to create an opening down to the source/drain electrodes.

Before the channel fabrication, the aqueous poly(3,4-ethylenedioxythiophene) polystyrene sulfonate (PEDOT:PSS) dispersion (Clevios™, PH 1000, Heraeus, Hanau, Germany) was mixed well with ethylene glycol (EG, 5 *v*/*v*%, Sigma-Aldrich, China), dodecyl benzene sulfonic acid (DBSA, 0.5 *v*/*v*%, Sigma-Aldrich, China), and 3-glycidoxypropyltrimethoxysilane (GOPS, 0.48 *v*/*v*%, Sigma-Aldrich, China), and filtered by a 0.45 μm filter to remove the undissolved solids. The PEDOT:PSS channel was then fabricated by spin-coating the PEDOT:PSS solution onto the device at a rate of 1500 rpm for 45 s. And the device was further annealed at 120 °C for 1 h, and immersed into the deionized water overnight to remove extra polystyrene sulfonate. Finally, the OECT device equipped with a PEDOT:PSS channel and a gold gate electrode was available by stripping off the sacrificial layer.

The PEDOT-PC and the hydroxyl-functionalized EDOT polymer (PEDOT-OH) coated gate electrodes were fabricated by electrochemically polymerizing EDOT-PC (synthesized following the previous approach reference [32]) and EDOT-OH (purchased from Sigma-Aldrich, China) on the Au disk electrodes of 2 mm diameter as shown in Figure 1b. The electropolymerization of the EDOT derivatives was performed using an Autolab PGSTAT128N potentiostat (Metrohm Autolab, Herisau, Switzerland) in a three-electrode electrochemical cell, with a Pt electrode (CH Instruments, Shanghai, China) as the counter electrode and an Ag/AgNO_3_ or Ag/AgCl as the reference electrode. The Au disk electrodes were used as the working electrodes. Before each measurement, the Ag/AgNO_3_ or Ag/AgCl reference electrodes were calibrated by the ferrocene/ferrocenium redox potential. The fabrication of the PEDOT-OH gate electrode was carried out by applying one cyclic potential scan from −0.6 V to 1.13 V versus Ag/AgCl onto the Au disk electrode in the aqueous 10 mM EDOT-OH solution supplemented with 100 mM LiClO_4_ and 50 mM sodium dodecyl sulfate (SDS). The PEDOT-PC gate electrode was prepared by further depositing ~20 nm thick PEDOT-PC onto ~26 nm thick PEDOT-OH (Figure S1) electrode in 10 mM EDOT-PC acetonitrile solution supplemented with 100 mM LiClO_4_ and 50 mM surfactant dioctyl sodium sulfosuccinate (DSS) under one cyclic potential scan ranging from −0.6 V to 1.025 V versus Ag/Ag^+^.

### 2.2. Water Contact Angle Measurement

The static water contact angles of PEDOT-OH and PEDOT-PC thin films were measured by a contact angle measurement system (Attention^®^ Theta Flex, Biolin Scientific, Gothenburg, Sweden) and recorded with a high-speed camera (T200/C) at ambient temperature (25 °C). Water was dispensed onto the surface of films set on a leveled base at a flow rate of 0.5 μL/s. The volume of the water drop was 4 μL. The analysis of contact angles from recorded videos was made using the OneAttension software. Each sample was measured three times to offer an average value.

### 2.3. Electrochemical Characterization

Cyclic voltammetry (CV) and impedance measurements were performed using a PalmSens 4 Potentiostat/Galvanostat in a three-electrode configuration. Platinum and an Ag/AgCl electrode (CH Instruments, Inc., China) were used as the counter and reference electrodes. For CV measurements, PEDOT-OH and PEDOT-PC-coated electrodes were used as working electrodes, and the CV curves were registered between −0.6 and 0.6 V at the scan rate of 100 mV s^−1^ in 0.1 M NaCl. Electrochemical impedance spectroscopy (EIS) was performed using a sinusoidal excitation signal of 10 mV amplitude with frequency varying from 0.1 to 100,000 Hz at an open circuit potential of electrodes in phosphate-buffered saline (PBS) buffer containing 5.0 mM [Fe(CN)_6_]^3/4−^ (1:1, mol/mol) as the redox couple. The samples were conditioned in PBS buffer with 5.0 mM [Fe(CN)_6_]^3/4−^ (1:1, mol/mol) for 10 s before the electrochemical impedance spectra (EIS) measurements.

### 2.4. Electrical Characterization of OECT Devices

Electrical characterization of the OECT device was performed with a Source Measure Unit (Keithley 2636B) in PBS or HEPES buffers. For output characteristics, the channel current *I_ds_* were measured as a function of drain voltage *V_ds_* sweeping from −0.8 to 0 V under a constant gate voltage *V_g_*, which varied between −0.4 and 0.8 V (step 0.2 V). The transfer curve was plotted as a function of the gate voltage *V_g_* sweeping from −0.4 to 1.2 V at *V_ds_* = −0.4 V. For each point, *I_ds_* values were measured with a delay of 0.1 s.

### 2.5. Quartz Crystal Microbalance Measurements (Protein Adsorption and CRP Binding)

Quartz Crystal Microbalance Measurements (QCM, Q-Sense AB system, Biolin Scientific) were performed to monitor the PEDOT films’ nonspecific and specific interaction with proteins at 25 °C. The films were deposited on the surface of a QSX 301 sensor crystal (Biolin Scientific) and then placed in the measurement chamber. Solutions of the biomolecules in PBS or 4-(2-Hydroxyethyl)piperazine-1-ethanesulfonic acid (HEPES) buffer were delivered continuously to the measurement chamber at a flow rate of 30 μL min^−1^ by an Ismatec ISM597D pump. With the interaction of the biomolecules in solution with the substrate on the sensor crystal, the corresponding changes in the sensor’s resonance frequency (Δ*f*), which is related to the attached mass, were recorded at a resolution of less than 1 s. If necessary, the pump was stopped for a few seconds to change the sample solutions without disturbing the QCM signal. The resonance frequencies were measured simultaneously at 5 MHz and its five harmonics (15, 25, 35, 45, 55, and 65 MHz). The changes in the frequency of the third overtone (n = 3, i.e., 15 MHz) are presented in the data. For the nonspecific protein adsorption test, the concentrations of the BSA and the fetal bovine serum (FBS) solution in PBS buffer are 1 mg mL^−1^ and 10% (volume ratio), respectively.

### 2.6. Detection of CRP

For CRP detection, the PEDOT-PC-coated gate electrodes were first dipped in CPR HEPES solution in the presence of Ca^2+^ for 30 min. Then, the gate electrodes were rinsed with Ca^2+^-containing HEPES buffer. The transfer characterization was measured by sweeping *V_g_* from −0.4 to 1.2 V at *V_ds_* = −0.4 V. For each point, *I_ds_* values were measured with a delay of 0.1 s. The gate voltage offset was calculated from the deviation of gate voltage under the maximum transconductance.

## 3. Results and Discussions

### 3.1. Design of PC-OECT Device

The fabrication process flow of the OECT array with three devices is shown in Figure 1a,b. The prepared OECT biosensor (PC-OECT) has a 20 μm × 40 μm PEDOT:PSS channel and a 2 mm PEDOT-PC gate electrode.

The specific detection of CRP for the PC-OECT sensor is illustrated in Figure 1c. The functionalization of PEDOT-PC imparts the gate of the OECT with super hydrophilicity and strong resistance to proteins due to the presence of zwitterionic phosphorylcholine groups, endowing the biosensor with anti-interference performance. Moreover, the phosphorylcholine group can specifically recognize CRP in the presence of calcium ions. Each protomer of CRP has a calcium-binding pocket in the phosphorylcholine binding domain, and the interaction of CRP with two calcium ions plays a significant role in phosphorylcholine recognition [38]. By combining the high transconductance of OECT with the antifouling and specific CRP binding capabilities of PEDOT-PC, this PC-OECT device can detect CRP with high sensitivity and excellent specificity, which are both critical in its point-of-care application in the real world.

### 3.2. Morphological, Electrochemical, and Electrical Properties of PEDOT-PC Gate

**Figure 2:**
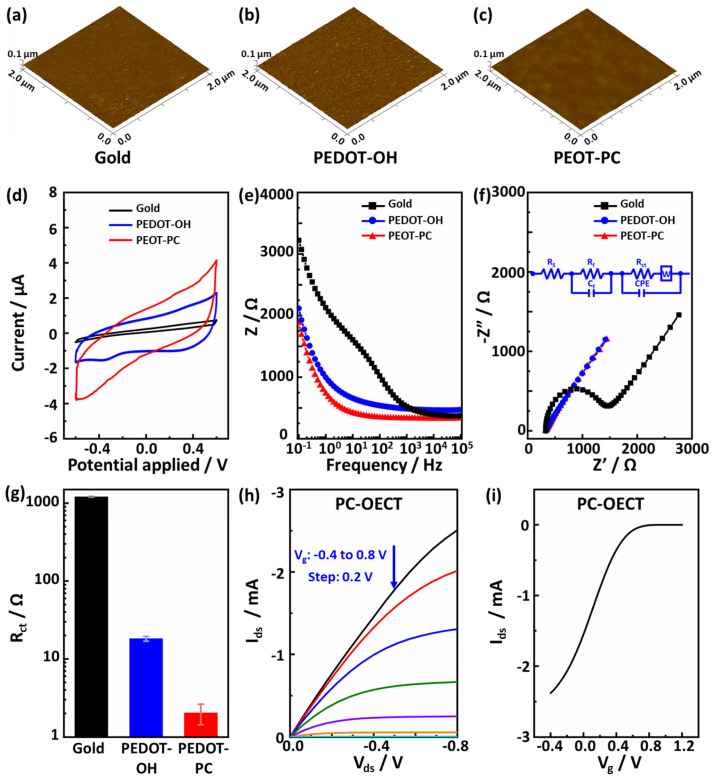
AFM images of the (**a**) gold, (**b**) PEDOT-OH, and (**c**) PEDOT-PC substrates. (**d**) Cyclic voltammograms profiles of gold, PEDOT-OH, and PEDOT-PC. (**e**) Bode plots and (**f**) Nyquist plots registered in PBS buffer with 10 mM [Fe(CN)_6_]^4−/3−^ for the gold, PEDOT-OH, and PEDOT-PC. The inset figure is the equivalent circuit model used to simulate these Nyquist plots. (**g**) The charge transfer resistance (*R_ct_*) value of the gold, PEDOT-OH, and PEDOT-PC electrode. Output curves (**h**) and Transfer curves (**i**) of PC-OECT in PBS buffer.

We used AFM to observe the surface morphologies of the gold, PEDOT-OH, and PEDOT-PC films. As shown in Figure 2a–c, the results indicated that these films are smooth, with the root mean square roughness of 2.51 nm, 3.65 nm, and 2.86 nm, respectively. To gain insights into the electrochemical properties and performance of the PEDOT-PC electrodes, we further carried out cyclic voltammetry (CV) and electrochemical impedance spectroscopy (EIS) measurements on the PEDOT-PC electrodes with the gold and PEDOT-OH electrodes as controls. Both the PEDOT-PC and PEDOT-OH electrodes presented a higher capacitance (Figure 2d) than the gold electrode (Figure 2d). It is thus reasonable to note in the Bode plots of the EIS data (Figure 2e) that both the PEDOT electrodes have a much lower impedance than gold electrodes in the low-frequency range, indicating their efficient charge transfer through solid–liquid interfaces. This could be attributed to the mixed ionic–electronic conductivity of PEDOT. PEDOT-PC exhibited slightly lower impedance than PEDOT-OH due to the enhanced ionic conductivity by incorporating zwitterions [39,40]. Zwitterions’ large dipole moment offers a large dielectric constant, shielding electrostatic attraction between anions and cations, thereby boosting ionic conductivity [41,42]. The Nyquist plots were generated and are shown in Figure 2f to elucidate the EIS data further. The equivalent circuit model with two parallel RC elements (inserted in Figure 2f) has been widely used to simulate conducting polymers’ electrochemical impedance spectroscopy (EIS) [43,44,45,46]. In this model, *R_s_*, *R_f_*, and *R_ct_* represent the resistance of solution resistance, film resistance, and charge transfer resistance, respectively; C_f_ represents the capacitance of the film; CPE is the constant phase element; and W is Warburg impedance. This model can effectively describe the contribution from the bulk of the conducting polymer and the conducting polymer interface with the electrolyte [47]. For the gold electrode, a classic equivalent circuit model with only one parallel RC element was used instead, as there is no film on its surface. These two models simulated all these impedance spectra within the frequency range from 0.1 Hz to 100,000 Hz, providing key insights into the electrode characteristics, as summarized in Table 1 and shown in Figure 2g.

As shown in Figure 2g, the charge transfer resistance (*R_ct_*) value of the PEDOT-PC electrode is three orders of magnitude smaller than that of the Au electrode, thanks to its volumetric electron–ion coupling. Furthermore, the *R_ct_* value of the PEDOT-PC electrode is only 11% of that of the PEDOT-OH electrode, plausibly attributed to the higher ionic conductivity of zwitterionic PEDOT-PC.

We further tested the electrical characteristics of the OECT device equipped with the PEDOT-PC gate electrodes (PC-OECT). Figure 2h shows the output characteristics of the PC-OECT, with negative bias at the drain (*V_ds_*) and *V_g_* varying from −0.4 V to 1.2 V. These features indicate a typical low-voltage operating mode of OECT, consistent with the depletion regime described by Bernards and Malliaras [48]. The time delay between sourcing *V_ds_* and *V_g_* and measuring *I_ds_* was set as 100 ms for registering each output curve, ensuring the drain current reached a steady state. The transfer characteristics are shown in Figure 2i for a drain voltage of −0.4 V. PEDOT:PSS contains a p-type PEDOT conducting polymer with the anions on the PSS to stabilize the holes. When a positive bias is applied on the gate, cations from the electrolyte enter the PEDOT:PSS film, compensate the anions on the PSS, and decrease the hole density on the PEDOT, which is reflected in the decrease in the drain current seen in Figure 2i [49].

### 3.3. Antifouling and Ca^2+^-Dependent Specific Interaction of PEDOT-PC Gate Electrode

**Figure 3:**
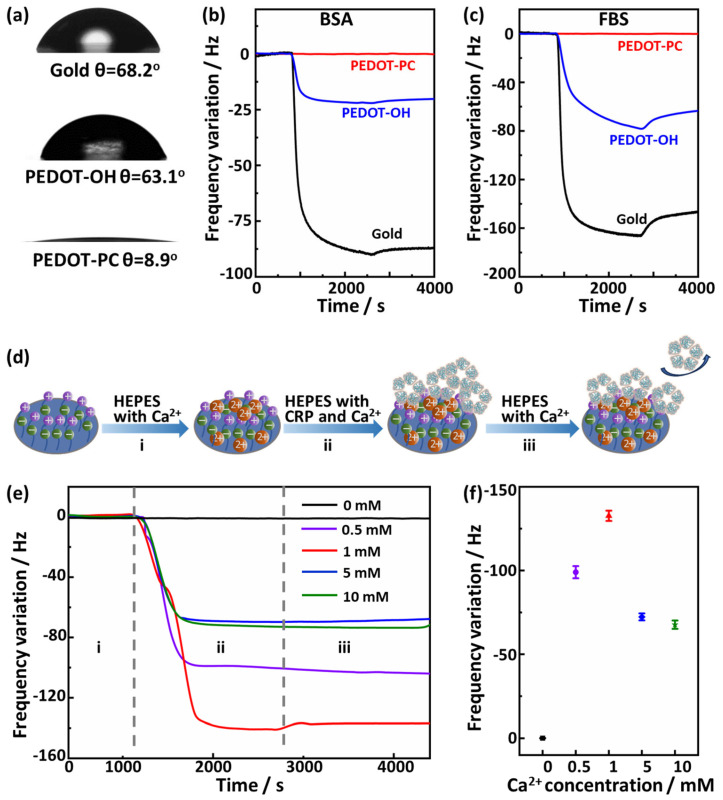
(**a**) Water contact angles of gold, PEDOT-OH, and PEDOT-PC. (**b**,**c**) Adsorption of proteins (BSA and FBS) to gold, PEDOT-OH, and PEDOT-PC. (**d**) A schematic diagram of QCM test process for CRP binding on PEDOT-PC surface. (**e**) Frequency variation in CRP (4 μg/mL) binding on PEDOT-PC films in HEPES buffer with different concentrations of Ca^2+^ ions. (**f**) Quantized frequency variation in CRP binding on PEDOT-PC films in HEPES buffer with different concentrations of Ca^2+^ ions.

It is ideal for sensing materials to resist the nonspecific interaction of biomolecules intrinsically, endowing the biosensor with excellent sensing stability and facilitating a specific detection without being interfered with by the biomolecules extensively existing in the biofluids. The PEDOT-PC electrode is super hydrophilic, as indicated by its small water contact angle of 8.9° (Figure 3a). Furthermore, it was found to be much more hydrophilic than the gold (*θ* = 68.2°) and PEDOT-OH (*θ* = 63.1°) electrodes. The hydrophilicity of these electrodes should be coherently correlated to their antifouling performances. As investigated by QCM experiments in Figure 3b,c, the gold and PEDOT-OH electrodes bound a large amount of the BSA and FBS proteins, resulting in a notable decrease in the QCM frequency. In sharp contrast, the zwitterionic PEDOT-PC electrode strongly resisted the protein biofouling, indicating its exceptional antifouling properties.

To evaluate the specific interaction of the PEDOT-PC electrode with CRP, we examined the CRP binding with the PEDOT-PC electrode under different concentrations of Ca^2+^ ions in a buffer solution. Usually, in the presence of calcium ions, the subunits of C-reactive proteins would bind calcium ions, forming binding sites for phosphate groups and, thus, enhancing the interaction between C-reactive proteins and phosphorylcholine groups. A QCM chip coated with PEDOT-PC was used for this binding test, as illustrated in Figure 3d.

In stage i, the chip frequency could reach an equilibrium value of HEPES buffer containing Ca^2+^. In stage ii, we exposed the PEDOT-PC-coated chip with the flow to the 4 μg/mL CRP HEPES buffer solution containing the same concentration of Ca^2+^. As shown in Figure 3e,f, there was no frequency drop if the HEPS buffer had no Ca^2+^. In contrast, a significant frequency drop occurred in the case of the HEPS buffer containing Ca^2+^, indicating that CRP can bind PEDOT-PC in the presence of Ca^2+^. In stage iii, the CRP molecules loosely absorbed were washed away by rinsing with HEPES buffer containing Ca^2+^. The QCM results revealed that the amount of the CRP molecules bound on the PEDOT-PC surface strongly depends on the Ca^2+^ concentration. It initially increased with the Ca^2+^ concentration and reached the maximum when the Ca^2+^ concentration increased to 1 mM. When the Ca^2+^ concentration was further increased, the amount of bound CRP molecules decreased with the increase in Ca^2+^ concentration. To verify whether the calcium ions interact with PEDOT-PC, the XPS spectra were also acquired for the PEDOT-PC films before and after exposure to the 1 mM Ca²⁺ solution (Figure S2). The binding energy of 2p electron of calcium (Ca) is typically located within 345–350 eV. However, as shown in Figure S2, the calcium XPS peak is absent in both PEDOT-PC films, indicating that the phosphorylcholine groups will not interact with the calcium ions if the C-reactive proteins are missing in the solution. These results clearly illustrate the necessity of Ca^2+^ ions to induce the specific recognition between CRP and phosphorylcholine groups. The interaction of two calcium ions with the CRP molecule plays a significant role in phosphorylcholine recognition [38].

### 3.4. Specific and Ultra-Sensitive CRP Detection 

**Figure 4:**
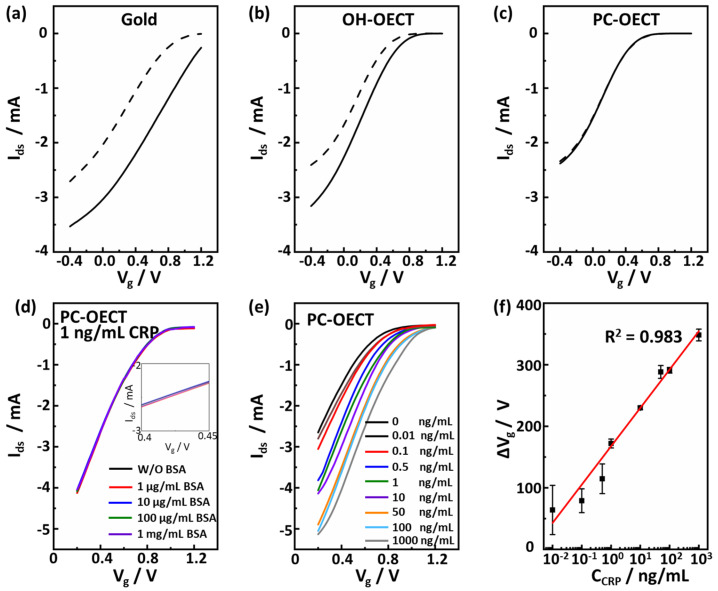
(**a**–**c**) Transfer curves of gold, OH-OECT, and PC-OECT in HEPES buffer before (solid line) and after (dash line) BSA solution soaking for 30 min. (**d**) Transfer curves of the PC-OECT in 1 ng/mL CRP HEPES (Ca^2+^) buffer with different concentrations of BSA interference. (**e**) Transfer curves of the PC-OECT with different CRP concentrations in HEPES (Ca^2+^) buffer. (**f**) The gate voltage offset for the PC-OECT after CRP binding with different CRP concentrations. Red line is the fitting line of the voltage offset.

To evaluate the anti-interference property of the OECT device, we measured the transfer curves of the devices gated by the gold (Gold-OECT), PEDOT-OH (OH-OECT), and PEDOT-PC (PC-OECT) electrodes were soaked in the HEPES buffers with and without 1 mg/mL BSA (Figure 4a–c). Both the gold-OECT and OH-OECT devices showed a significant voltage offset in transfer characteristics after the gate electrodes soaked in the BSA solution, indicating that the BSA molecules were nonspecifically bound on the gate electrodes and distinctively affected the transfer curves. In sharp contrast, no voltage offset was observed in the transfer curve for the PC-OECT device after the PEDOT-PC gate soaked in the BSA solution, indicating that the strong protein resistance of PEDOT-PC significantly depressed the nonspecific binding of BSA molecules.

Detection specificity is much more critical in the clinical application of the real world, especially for a portable and easy-to-operate device targeting point-of-care CRP detection, as clinical samples—mostly human biofluids—contain a lot of proteins, which nonspecifically bind on the electrodes and distort the detection results. Therefore, we used the CRP HEPS solution containing BSA to simulate a clinical sample and evaluated the specificity of the PC-OECT device in detecting 1 ng/mL CRP molecules by gradually increasing the BSA concentration. As shown in Figure 4d, the transfer curve presented almost no change, even though the BSA concentration rose to 1 mg/mL, which is six orders of magnitude higher than the biomarker concentration. In detail, the 1 mg/mL BSA only led to a negligible voltage offset (~6 mV), indicating excellent anti-interference and detection specificity of the PC-OEDT device.

Based on these results, we further assessed the CRP detection of the PC-OECT device in a wide range of CRP concentrations. The HEPES buffer solutions with different CRP concentrations (ranging from 0.01 ng/mL to 1000 ng/mL) and 1 mM/mL calcium ion were prepared for the CRP measurement. After the PEDOT-PC gate was soaked in the samples, the transfer curves were registered to measure the corresponding gate voltage offsets induced by the specific interaction of CRP molecules with gates (Figure 4e). The CRP molecules binding on the PEDOT-PC gates would reduce the potential value working effectively on the channel due to their negative charge in the HEPES buffer, consequently shifting the transfer curve to the higher potential [4]. The transfer curve presented a voltage offset of 64 mV at a CRP concentration of 0.01 ng/mL, whereas the voltage offset at 1000 ng/mL was approximately 348 mV (Figure 4e,f). The gate voltage offset was noted to be linearly dependent on the logarithm of the CRP concentration in the concentration region from 0.01 ng/mL to 1000 ng/mL, with a linear coefficient (*R*^2^) of 0.983 (Figure 4f). The present detection limit (LOD) for the CRP molecules was found to be 0.01 ng/mL, i.e., 10 pg/mL.

We further conducted a comprehensive comparison between our CRP detection method with other label-free electrochemical techniques, such as transistors, voltammetric sensors, ASV sensors, impedance sensors, and DPV sensors, as shown in Table 2. It is worth emphasizing that the LOD of our device is either smaller than or, at the very least, comparable to the LODs of previously established electrochemical methods. Moreover, this PEDOT-PC-gated OECT device could detect the CRP molecules directly without the nonspecific binding blocking, as the PEDOT-PC gate electrode is intrinsically antifouling. However, the earlier electrochemical devices need BSA blocking to remove the surface nonspecific binding, thus, improving their detection specificity [4,11,14]. However, significant cross-reactivity was reported frequently for BSA blocking because of the bovine IgG contamination of commercial BSA [50,51,52]. Additionally, its batch-to-batch variability and its steric hindrance to antigen–antibody interactions and endogenous enzyme activity are also claimed to be major drawbacks of BSA blocking [53,54,55,56,57]. Moreover, BSA and other blocking agents are normally electrically charged, and their binding would dramatically change the electrochemical properties of gates/channels and, thus, the sensing performance of transistors. This, in combination with the batch variability of BSA, makes it necessary to conduct calibration after BSA blocking. We considered that the PEDOT-PC-gated OECT biosensor could address these issues well since the PEDOT-PC electrode intrinsically resists the nonspecific binding of biomolecules and does not need BSA blocking, and makes the scalable usage of sensors among inexperienced persons possible.

## 4. Conclusions

In this work, we fabricated a PEDOT-PC-gated OECT biosensor targeting detecting ultra-trace CRP molecules in a complex biological environment. This PEDOT-PC electrode could completely prevent BSA and FBS binding while specifically interacting with CRP molecules in the presence of calcium ions. Unlike those of the gold and PEDOT-OH-gated devices, the transfer curves of the PEDOT-PC-gated device did not change when exposed to a high-concentration BSA solution. It thus ensured an excellent detection specificity toward ultra-trace CRP molecules in the presence of 1 mg/mL BSA. The PEDOT-PC-gated OECT device finally demonstrated a sensitive response to CRP molecules ranging from 0.01 ng/mL to 1000 ng/mL with a detection limit of 0.01 ng/mL. We envisioned that this PEDOT-PC-gated OECT platform could alleviate concern about poor antibody stability and BSA blocking inconstancy and potentially offer a sensitive and easy-to-operate method for detecting CRP molecules in the real world; however, our PC-OECT devices must undergo rigorous validation using clinical samples and be subjected to a comparative analysis against existing techniques before their application in real-world scenarios. This validation process is crucial to establish the reliability and accuracy of the device’s performance. Moreover, the PC-OECT device remains confronted by one prominent limitation that stems from its reliance on the presence of Ca^2+^ ions for its operational functionality. To pave the way for applying the PC-OECT device to a wide array of scenarios, future research endeavors should be concentrated on refining its technical aspects to enhance its adaptability across diverse conditions.

## Figures and Tables

**Table 1:** Electrochemical parameters of the Au and PEDOT electrodes by simulating the EIS data of PEDOT electrodes using the equivalent circuit models.

Sample	*R_s_* (Ω)	*R_f_* (Ω)	*C_f_* (μF)	*R_ct_* (Ω)	*CPE*	*W* (Kσ)	*χ* ^2^
Gold	202 ± 3.6	/	/	1207 ± 12.5	2.1 ± 0.05 μT; 0.790 ± 0.004 Φ	1.1 ± 0.02	0.0005
PEDOT-OH	208 ± 1.6	5.94 ± 1.2	2.87 ± 0.5	18.2 ± 1.3	5.3 ± 1.15 μT; 0.514 ± 0.006 Φ	1.1 ± 0.03	0.0005
PEDOT-PC	211 ± 2.2	23.7 ± 3.6	4.82 ± 0.2	2.03 ± 0.6	3.6 ± 0.69 μT; 0.783 ± 0.03 Φ	1.0 ± 0.07	0.0006

**Table 2:** Summary of transistor-based approaches for CRP detection (Abbreviation: FET = Field-Effect Transistor; pTHMMAA = N-[tris(hydroxymethyl) methyl] acrylamide-lipoic acid conjugate; DPh-BBTNDT = 2,10-diphenylbis[1]benzothieno [2,3-d;2′,3′-d′]naphtho [2,3b;6,7-b′] dithiophene; Anodic stripping voltammetry = ASV.

Sensor Type	Functionalized Interface	Sensing Unit	Limit of Detection (LOD)	Detection Range	Linear Range	Strategy to Ensure Specificity	References
OFET	Channel	Antibody	1 μg/mL	1 μg/mL–50 μg/mL	1 μg/mL–50 μg/mL	/	[12]
FET	Gate Channel (AlGaN/GaN)	Antibody	10 ng/mL	10 ng/mL–1000 ng/mL	/	/	[58]
OFET	Channel	Antibody	2 pM (0.23 ng/mL)	4 pM–2 μM (0.46 ng/mL–0.23 mg/mL)	4 pM–2 μM (0.46 ng/mL–0.23 mg/mL)	pTHMMAA	[13]
OFET	Gate Channel (DPh-BBTNDT)	Antibody	100 ng/mL	100 ng/mL–1 μg/mL	500 ng/mL–1 μg/mL	BSA block	[4]
FET	Channel	Antibody	0.1 ng/mL	0.1 ng/mL–100 μg/mL	0.1 ng/mL–100 μg/mL	BSA block	[11]
OECT	Gate	Antibody	10 pg/mL	10 pg/mL–0.2 mg/mL	10 pg/mL–0.2 mg/mL	BSA block	[14]
Voltammetric sensor	Electrode	Antibody	3 ng/mL	1 ng/mL–10 μg/mL	/	BSA block	[59]
ASV sensor	Electrode	Antibody	0.05 ng/mL	0.2 ng/mL–100 ng/mL	0.2 ng/mL–100 ng/mL	BSA block	[60]
Voltammetric and impedance sensor	Electrode	Antibody	11 ng/mL	50 ng/mL–5 μg/mL	/	/	[61]
Impedance sensor	Electrode	Antibody	60 pg/mL	1–1000 ng/mL	1–1000 ng/mL	BSA block	[62]
OECT	Gate	PEDOT-PC	10 pg/mL	10 pg/mL–1 μg/mL	10 pg/mL–1 μg/mL	Intrinsically antifouling	This work

## Data Availability

The data presented in this study are available on request from the corresponding author.

## Supplementary Materials

The following supporting information can be downloaded at: https://www.mdpi.com/article/10.3390/polym15183739/s1, Figure S1: Surface topographies of (a) the PEDOT-OH film and (b) the PEDOT-PC film deposited on the PEDOT-OH film (scan range: 5 μm × 5 μm) and height profiles of the scratches of (c) the PEDOT-OH film and (d) the PEDOT-PC film deposited on the PEDOT-OH film; Figure S2: XPS spectra of the PEDOT-PC and PEDOT-PC after exposure to the 1 mM Ca²⁺ solution.

## References

[1] Gewurz H. (1982). Biology of C-Reactive Protein and the Acute Phase Response. Hosp. Pract..

[2] Pepys M.B., Hirschfield G.M., Tennent G.A., Gallimore J.R., Kahan M.C., Bellotti V., Hawkins P.N., Myers R.M., Smith M.D., Polara A. (2006). Targeting C-reactive protein for the treatment of cardiovascular disease. Nature.

[3] Clyne B., Olshaker J.S. (1999). The C-reactive protein. J. Emerg. Med..

[4] Ji X., Zhou P., Zhong L., Xu A., Tsang A.C.O., Chan P.K.L. (2018). Smart Surgical Catheter for C-Reactive Protein Sensing Based on an Imperceptible Organic Transistor. Adv. Sci..

[5] Dorraki M., Fouladzadeh A., Salamon S.J., Allison A., Coventry B.J., Abbott D. (2018). On detection of periodicity in C-reactive protein (CRP) levels. Sci. Rep..

[6] Musunuru K., Kral B.G., Blumenthal R.S., Fuster V., Campbell C.Y., Gluckman T.J., A Lange R., Topol E.J., Willerson J.T., Desai M.Y. (2008). The use of high-sensitivity assays for C-reactive protein in clinical practice. Nat. Clin. Pract. Cardiovasc. Med..

[7] Pepys M.B., Hirschfield G.M. (2003). C-reactive protein: A critical update. J. Clin. Investig..

[8] Wu T.-L., Tsao K.-C., Chang C.P.-Y., Li C.-N., Sun C.-F., Wu J.T. (2002). Development of ELISA on microplate for serum C-reactive protein and establishment of age-dependent normal reference range. Clin. Chim. Acta.

[9] Härmä H., Toivonen J., Soini J.T., Hänninen P., Parak W.J. (2011). Time-Resolved Fluorescence Immunoassay for C-Reactive Protein Using Colloidal Semiconducting Nanoparticles. Sensors.

[10] Huang L., Liao T., Wang J., Ao L., Su W., Hu J. (2018). Brilliant Pitaya-Type Silica Colloids with Central-Radial and High-Density Quantum Dots Incorporation for Ultrasensitive Fluorescence Immunoassays. Adv. Funct. Mater..

[11] Justino C.I., Freitas A.C., Amaral J.P., Rocha-Santos T.A., Cardoso S., Duarte A.C. (2013). Disposable immunosensors for C-reactive protein based on carbon nanotubes field effect transistors. Talanta.

[12] Palazzo G., De Tullio D., Magliulo M., Mallardi A., Intranuovo F., Mulla M.Y., Favia P., Vikholm-Lundin I., Torsi L. (2015). Detection Beyond Debye’s Length with an Electrolyte-Gated Organic Field-Effect Transistor. Adv. Mater..

[13] Magliulo M., De Tullio D., Vikholm-Lundin I., Albers W.M., Munter T., Manoli K., Palazzo G., Torsi L. (2016). Label-free C-reactive protein electronic detection with an electrolyte-gated organic field-effect transistor-based immunosensor. Anal. Bioanal. Chem..

[14] Li M., Shu Q., Qing X., Wu J., Xiao Q., Jia K., Wang X., Wang D. (2023). Boron nitride-mediated semiconductor nanonetwork for an ultralow-power fibrous synaptic transistor and C-reactive protein sensing. J. Mater. Chem. C.

[15] Burtscher B., Urbina P.A.M., Diacci C., Borghi S., Pinti M., Cossarizza A., Salvarani C., Berggren M., Biscarini F., Simon D.T. (2021). Sensing Inflammation Biomarkers with Electrolyte-Gated Organic Electronic Transistors. Adv. Healthc. Mater..

[16] Rivnay J., Inal S., Salleo A., Owens R.M., Berggren M., Malliaras G.G. (2018). Organic electrochemical transistors. Nat. Rev. Mater..

[17] Wang N., Yang A., Fu Y., Li Y., Yan F. (2019). Functionalized Organic Thin Film Transistors for Biosensing. Acc. Chem. Res..

[18] Lin P., Yan F. (2012). Organic Thin-Film Transistors for Chemical and Biological Sensing. Adv. Mater..

[19] Berggren M., Crispin X., Fabiano S., Jonsson M.P., Simon D.T., Stavrinidou E., Tybrandt K., Zozoulenko I. (2019). Ion Electron–Coupled Functionality in Materials and Devices Based on Conjugated Polymers. Adv. Mater..

[20] Kim J.H., Kim S., Kim G., Yoon M. (2020). Designing Polymeric Mixed Conductors and Their Application to Electrochemical-Transistor-Based Biosensors. Macromol. Biosci..

[21] Romele P., Ghittorelli M., Kovács-Vajna Z.M., Torricelli F. (2019). Ion buffering and interface charge enable high performance electronics with organic electrochemical transistors. Nat. Commun..

[22] Campana A., Cramer T., Simon D.T., Berggren M., Biscarini F. (2014). Electrocardiographic Recording with Conformable Organic Electrochemical Transistor Fabricated on Resorbable Bioscaffold. Adv. Mater..

[23] Lee W., Kobayashi S., Nagase M., Jimbo Y., Saito I., Inoue Y., Yambe T., Sekino M., Malliaras G.G., Yokota T. (2018). Nonthrombogenic, stretchable, active multielectrode array for electroanatomical mapping. Sci. Adv..

[24] Guo K., Wustoni S., Koklu A., Díaz-Galicia E., Moser M., Hama A., Alqahtani A.A., Ahmad A.N., Alhamlan F.S., Shuaib M. (2021). Rapid single-molecule detection of COVID-19 and MERS antigens via nanobody-functionalized organic electrochemical transistors. Nat. Biomed. Eng..

[25] Rushton A.J., Nteliopoulos G., Shaw J.A., Coombes R.C. (2021). A Review of Circulating Tumour Cell Enrichment Technologies. Cancers.

[26] Romeo A., Tarabella G., D’angelo P., Caffarra C., Cretella D., Alfieri R., Petronini P.G., Iannotta S. (2015). Drug-induced cellular death dynamics monitored by a highly sensitive organic electrochemical system. Biosens. Bioelectron..

[27] Ramuz M., Hama A., Huerta M., Rivnay J., Leleux P., Owens R.M. (2014). Combined Optical and Electronic Sensing of Epithelial Cells Using Planar Organic Transistors. Adv. Mater..

[28] Lin P., Yan F., Yu J., Chan H.L.W., Yang M. (2010). The Application of Organic Electrochemical Transistors in Cell-Based Biosensors. Adv. Mater..

[29] Wang J., Ye D., Meng Q., Di C., Zhu D. (2020). Advances in Organic Transistor-Based Biosensors. Adv. Mater. Technol..

[30] Yu J., Yang A., Wang N., Ling H., Song J., Chen X., Lian Y., Zhang Z., Yan F., Gu M. (2021). Highly sensitive detection of caspase-3 activity based on peptide-modified organic electrochemical transistor biosensors. Nanoscale.

[31] Qian S., Lin H.-A., Pan Q., Zhang S., Zhang Y., Geng Z., Wu Q., He Y., Zhu B. (2023). Chemically revised conducting polymers with inflammation resistance for intimate bioelectronic electrocoupling. Bioact. Mater..

[32] Zhu B., Luo S.-C., Zhao H., Lin H.-A., Sekine J., Nakao A., Chen C., Yamashita Y., Yu H.-H. (2014). Large enhancement in neurite outgrowth on a cell membrane-mimicking conducting polymer. Nat. Commun..

[33] Thompson D., Pepys M.B., Wood S.P. (1999). The physiological structure of human C-reactive protein and its complex with phosphocholine. Structure.

[34] Park J., Kurosawa S., Watanabe J., Ishihara K. (2004). Evaluation of 2-Methacryloyloxyethyl Phosphorylcholine Polymeric Nanoparticle for Immunoassay of C-Reactive Protein Detection. Anal. Chem..

[35] Wu J.-G., Wei S.-C., Chen Y., Chen J.-H., Luo S.-C. (2018). Critical Study of the Recognition between C-Reactive Protein and Surface-Immobilized Phosphorylcholine by Quartz Crystal Microbalance with Dissipation. Langmuir.

[36] Goda T., Ishihara K., Miyahara Y. (2015). Critical update on 2-methacryloyloxyethyl phosphorylcholine (MPC) polymer science. J. Appl. Polym. Sci..

[37] Goda T., Toya M., Matsumoto A., Miyahara Y. (2015). Poly(3,4-ethylenedioxythiophene) Bearing Phosphorylcholine Groups for Metal-Free, Antibody-Free, and Low-Impedance Biosensors Specific for C-Reactive Protein. ACS Appl. Mater. Interfaces.

[38] Christopeit T., Gossas T., Danielson U.H. (2009). Characterization of Ca2+ and phosphocholine interactions with C-reactive protein using a surface plasmon resonance biosensor. Anal. Biochem..

[39] Tiyapiboonchaiya C., Pringle J.M., Sun J., Byrne N., Howlett P.C., MacFarlane D.R., Forsyth M. (2004). The zwitterion effect in high-conductivity polyelectrolyte materials. Nat. Mater..

[40] Rebollar L., Panzer M.J. (2019). Zwitterionic Copolymer-Supported Ionogel Electrolytes: Impacts of Varying the Zwitterionic Group and Ionic Liquid Identities. ChemElectroChem.

[41] Mei W., Rothenberger A.J., Bostwick J.E., Rinehart J.M., Hickey R.J., Colby R.H. (2021). Zwitterions Raise the Dielectric Constant of Soft Materials. Phys. Rev. Lett..

[42] Xu H., Huang L., Li W., Gu S., Zeng D., Zhang Y., Sun Y., Cheng H. (2022). Shielding the electrostatic attraction by design of zwitterionic single ion conducting polymer electrolyte with high dielectric constant. J. Membr. Sci..

[43] Hafaid I., Chebil S., Korri-Youssoufi H., Bessueille F., Errachid A., Sassi Z., Ali Z., Abdelghani A., Jaffrezic-Renault N. (2010). Effect of electrical conditions on an impedimetric immunosensor based on a modified conducting polypyrrole. Sens. Actuators B Chem..

[44] Nessark F., Eissa M.M., Baraket A., Zine N., Nessark B., Zouaoui A., Bausells J., Errachid A. (2020). Capacitance Polypyrrole-based Impedimetric Immunosensor for Interleukin-10 Cytokine Detection. Electroanalysis.

[45] Ding Z., Zhang Q., Chen Y., Liu G., Xin X., He H., Cai B., Wu J., Yao X. (2019). PEDOT-PSS coated VS_2_ nanosheet anodes for high rate and ultrastable lithium-ion batteries. New J. Chem..

[46] Pan Q., Wu Q., Sun Q., Zhou X., Cheng L., Zhang S., Yuan Y., Zhang Z., Ma J., Zhang Y. (2022). Biomolecule-friendly conducting PEDOT interface for long-term bioelectronic devices. Sens. Actuators B Chem..

[47] Jaffrezic-Renault N. (2013). Label-Free Affinity Biosensors Based on Electrochemical Impedance Spectroscopy. Microelectrode Biosens..

[48] Bernards D.A., Malliaras G.G. (2007). Steady-State and Transient Behavior of Organic Electrochemical Transistors. Adv. Funct. Mater..

[49] Khodagholy D., Gurfinkel M., Stavrinidou E., Leleux P., Herve T., Sanaur S., Malliaras G.G. (2011). High speed and high density organic electrochemical transistor arrays. Appl. Phys. Lett..

[50] Andersen D.C., Koch C., Jensen C.H., Skjødt K., Brandt J., Teisner B. (2004). High Prevalence of Human Anti-bovine IgG Antibodies as the Major Cause of False Positive Reactions in Two-Site Immunoassays Based on Monoclonal Antibodies. J. Immunoass. Immunochem..

[51] Willman J.H., Martins T.B., Jaskowski T.D., Hill H.R., Litwin C.M. (1999). Heterophile Antibodies to Bovine and Caprine Proteins Causing False-Positive Human Immunodeficiency Virus Type 1 and Other Enzyme-Linked Immunosorbent Assay Results. Clin. Diagn. Lab. Immunol..

[52] Miura H., Kitano M., Yoneyama A., Kuwahara A., Moriyama K., Kitajima S. (2005). IgG heterophile antibody causes false positivity for CA19-9, which is overcome with bovine immunoglobulin. Rinsho Byori. Jpn. J. Clin. Pathol..

[53] Ambroz K.L.H., Zhang Y., Schutz-Geschwender A., Olive D.M. (2008). Blocking and detection chemistries affect antibody performance on reverse phase protein arrays. Proteomics.

[54] Xiao Y., Isaacs S.N. (2012). Enzyme-linked immunosorbent assay (ELISA) and blocking with bovine serum albumin (BSA)—Not all BSAs are alike. J. Immunol. Methods.

[55] DenHollander N., Befus D. (1989). Loss of antigens from immunoblotting membranes. J. Immunol. Methods.

[56] Craig W.Y., Poulin S.E., Collins M.F., Ledue T.B., Ritchie R.F. (1993). Background staining in immunoblot assays reduction of signal caused by cross-reactivity with blocking agents. J. Immunol. Methods.

[57] Craig W.Y., E Poulin S., Nelson C.P., Ritchie R.F. (1994). ELISA of IgG antibody to oxidized low-density lipoprotein: Effects of blocking buffer and method of data expression. Clin. Chem..

[58] Lee H.H., Bae M., Jo S.H., Shin J.K., Son D.H., Won C.H., Jeong H.M., Lee J.H., Kang S.W. (2015). AlGaN/GaN High Electron Mobility Transistor-Based Biosensor for the Detection of C-Reactive Protein. Sensors.

[59] Petruzzi L., Maier T., Ertl P., Hainberger R. (2022). Quantitative detection of C-reactive protein in human saliva using an electrochemical lateral flow device. Biosens. Bioelectron. X.

[60] Kokkinos C., Prodromidis M., Economou A., Petrou P., Kakabakos S. (2015). Disposable integrated bismuth citrate-modified screen-printed immunosensor for ultrasensitive quantum dot-based electrochemical assay of C-reactive protein in human serum. Anal. Chim. Acta.

[61] Gupta R.K., Periyakaruppan A., Meyyappan M., Koehne J.E. (2014). Label-free detection of C-reactive protein using a carbon nanofiber based biosensor. Biosens. Bioelectron..

[62] Yagati A.K., Pyun J.-C., Min J., Cho S. (2016). Label-free and direct detection of C-reactive protein using reduced graphene oxide-nanoparticle hybrid impedimetric sensor. Bioelectrochemistry.

