# Supplementary material for: Phosphorylcholine-Functionalized PEDOT-Gated Organic Electrochemical Transistor Devices for Ultra-Specific and Sensitive C-Reactive Protein Detection
Source: Polymers (Basel). 2023 Sep 12;15(18):3739. doi: 10.3390/polym15183739 (PMC10535691; doi:10.3390/polym15183739)
Supplement: Supplementary file 1 [file polymers-15-03739-s001.zip › polymers-2555630-supplementary.pdf]

polymers

MDPI

# Supplementary Material: Phosphorylcholine-Functionalized PEDOT-Gated Organic Electrochemical Transistor Devices for Ultra-Specific and Sensitive C-Reactive Protein Detection

**Sihao Qian, Shouyan Zhang, Danni Chen, Jun Wang, Wei Wu, Shuhua Zhang, Zhi Geng, Yong He, and Bo Zhu**

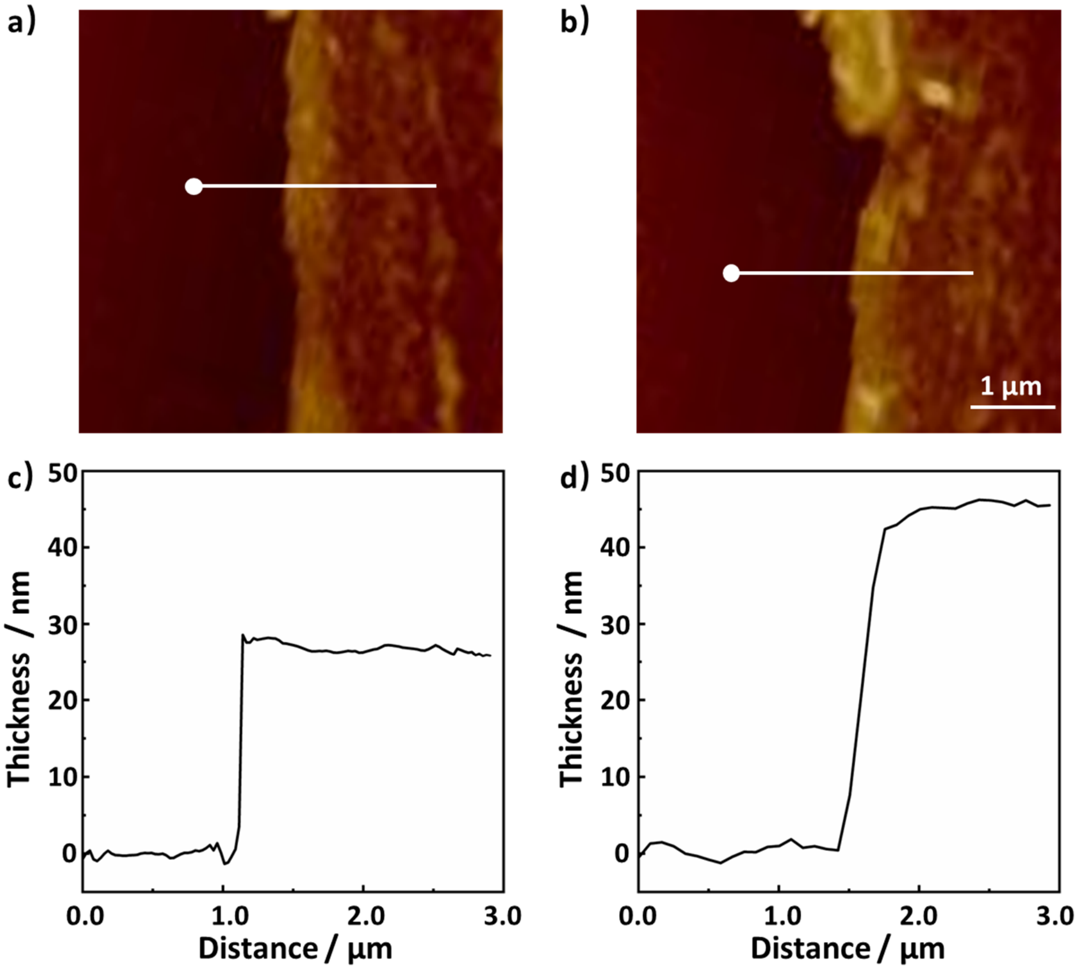


**Figure S1.** Surface topographies of (**a**) the PEDOT-OH film and (**b**) the PEDOT-PC film deposited on the PEDOT-OH film (scan range: 5 µm × 5 µm) and height profiles of the scratches of (**c**) the PEDOT-OH film and (**d**) the PEDOT-PC film deposited on the PEDOT-OH film.

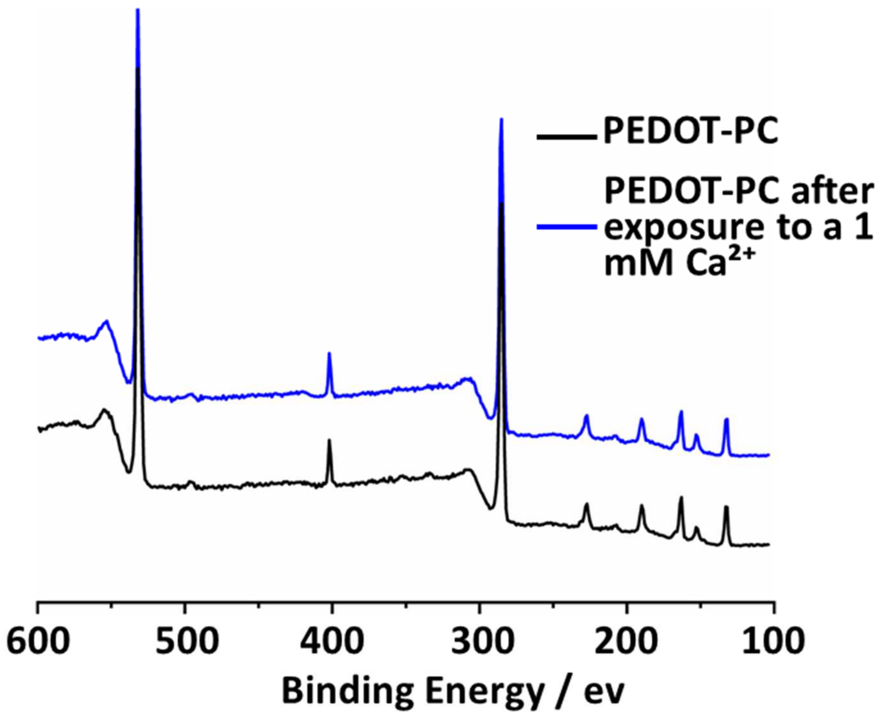


**Figure S2.** XPS spectra of the PEDOT-PC and PEDOT-PC after exposure to the 1 mM $Ca^{2+}$ solution.
